# Unravelling the pH-dependence of a molecular photocatalytic system for hydrogen production†
†Electronic supplementary information (ESI) available. See DOI: 10.1039/c5sc01349f



**DOI:** 10.1039/c5sc01349f

**Published:** 2015-05-28

**Authors:** Anna Reynal, Ernest Pastor, Manuela A. Gross, Shababa Selim, Erwin Reisner, James R. Durrant

**Affiliations:** a Department of Chemistry , Imperial College London , Exhibition Road , London SW7 2AZ , UK . Email: j.durrant@imperial.ac.uk; b School of Chemistry , Newcastle University , Newcastle Upon Tyne , NE1 7RU , UK . Email: anna.reynal@ncl.ac.uk; c Christian Doppler Laboratory for Sustainable SynGas Chemistry , Department of Chemistry , University of Cambridge , Lensfield Road , Cambridge CB2 1EW , UK . Email: reisner@ch.cam.ac.uk

## Abstract

Photocatalytic systems for the reduction of aqueous protons are strongly pH-dependent, but the origin of this dependency is still not fully understood. We have studied the effect of different degrees of acidity on the electron transfer dynamics and catalysis taking place in a homogeneous photocatalytic system composed of a phosphonated ruthenium tris(bipyridine) dye (**RuP**) and a nickel bis(diphosphine) electrocatalyst (**NiP**) in an aqueous ascorbic acid solution. Our approach is based on transient absorption spectroscopy studies of the efficiency of photo-reduction of **RuP** and **NiP** correlated with pH-dependent photocatalytic H_2_ production and the degree of catalyst protonation. The influence of these factors results in an observed optimum photoactivity at pH 4.5 for the **RuP**–**NiP** system. The electron transfer from photo-reduced **RuP** to **NiP** is efficient and independent of the pH value of the medium. At pH <4.5, the efficiency of the system is limited by the yield of **RuP** photo-reduction by the sacrificial electron donor, ascorbic acid. At pH >4.5, the efficiency of the system is limited by the poor protonation of **NiP**, which inhibits its ability to reduce protons to hydrogen. We have therefore developed a rational strategy utilising transient absorption spectroscopy combined with bulk pH titration, electrocatalytic and photocatalytic experiments to disentangle the complex pH-dependent activity of the homogenous **RuP**–**NiP** photocatalytic system, which can be widely applied to other photocatalytic systems.

## Abstract 2

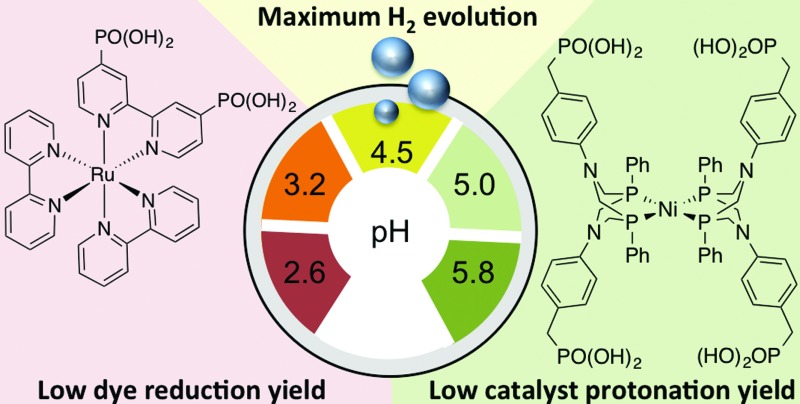
The electron-donating ability of the sacrificial agent and the protonation of the catalyst determine the optimum pH for hydrogen production.

## Introduction

The photochemical production of H_2_ from water is a rapidly expanding research field that aims to store solar energy in a chemical fuel.^1^ From the viewpoint of sustainability and economic viability, this proton reduction reaction should be carried out in aqueous conditions and use stable and Earth abundant materials.^2^ Current investigations for solar H_2_ synthesis include molecular dyes and electrocatalysts based on nickel, iron and cobalt, either in solution or immobilised onto the surface of a semiconductor.^3–12^ These photocatalytic systems typically require the use of sacrificial chemical reductants to provide the electrons to regenerate the oxidised dye following proton reduction.

The efficiency of H_2_ evolving photo- and electrocatalytic systems is typically strongly pH dependent.^13–16^ Understanding the origins of this pH dependence is critical to guide further system development and optimisation. In particular, it is essential to determine whether such pH dependencies derive from the availability of protons to the molecular catalyst, from the function of the molecular light-harvesting unit or from the sacrificial electron donor.

**Fig. 1:**
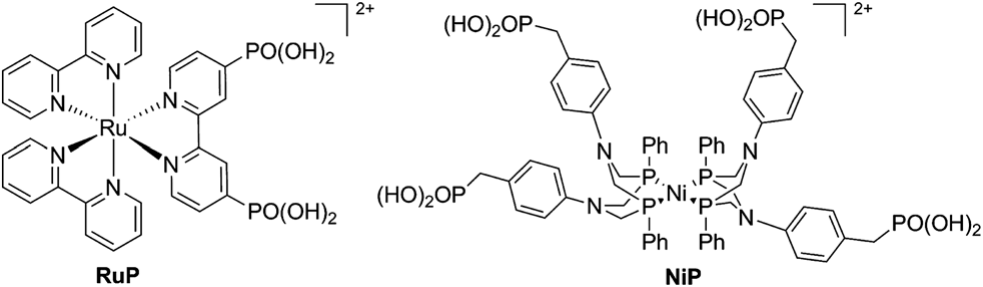
Molecular structures of the phosphonated ruthenium dye (**RuP**) and the nickel H_2_ evolution catalyst (**NiP**). The bromide counter ions have been omitted for clarity.

**Scheme 1:**
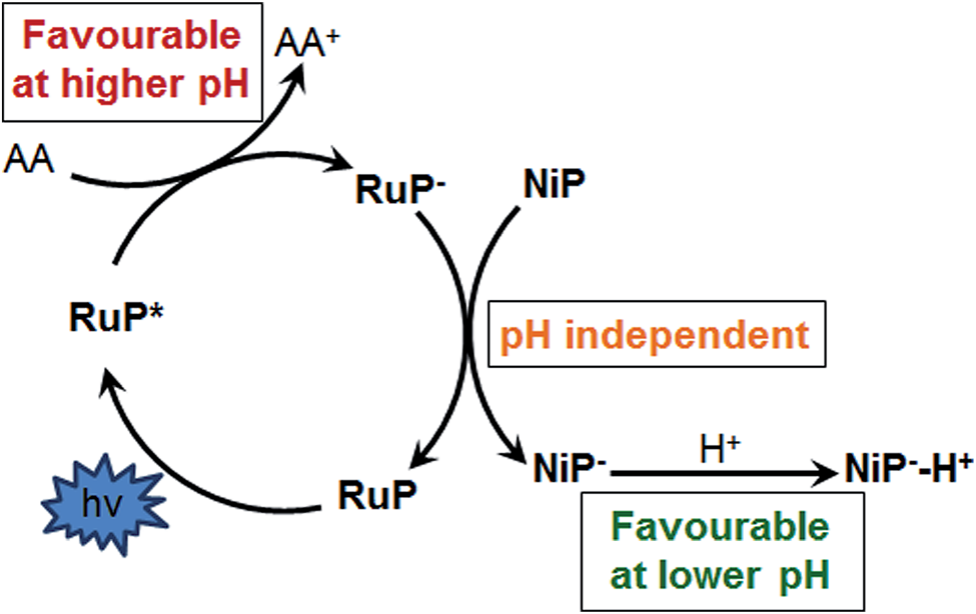
Schematic representation of the reductive electron transfer mechanism between **RuP** and **NiP** in the presence of ascorbic acid as sacrificial electron donor.

We have recently reported a homogeneous photocatalytic system based on a molecular ruthenium photosensitiser (**RuP**) and a nickel catalyst (**NiP**) capable of producing H_2_ in pure water with a quantum efficiency near 10% in the presence of ascorbic acid (AA) as a sacrificial electron donor (Fig. 1).^17^ In this system, the electron transfer from the photoreduced dye (**RuP^–^**) to **NiP** takes place following reductive quenching of the photoexcited dye in the presence of the sacrificial agent, AA (Scheme 1). Under visible light irradiation, optimum performance of this photocatalytic system was observed at pH 4.5. In contrast, when used as an electrocatalyst, the proton reduction efficiency of the **NiP** catalyst was observed to increase towards more acidic pH.^17^ This pH dependence is typical of this type of nickel-based molecular electrocatalysts, and has been attributed to the presence of pendant amines with low p*K*_a_, which are thought to act as a proton relay between the solvent and the metal centre.^13,18–20^

Studies reporting the dependence of H_2_ evolution on the acidity of the aqueous media for molecular photocatalytic systems have typically focused on the overall system efficiency as a function of pH.^13–15,21^ Reaction mechanisms, where studied, have been addressed through theoretical calculations and experimental techniques such as nuclear magnetic resonance spectroscopy, electrochemistry and steady state spectroscopy;^7,22–24^ and to a lesser extent, time-resolved absorption spectroscopy.^15,24–34^ Herein, we report on the influence of the solution acidity on the formation of the photo-reduced **RuP^–^** species, the electron transfer kinetics between the optically active **RuP^–^** and **NiP**, as well as the pH dependence of H_2_ evolution observed in electrochemical and bulk photocatalytic experiments. We have employed transient absorption spectroscopy, combined with electrochemical experiments, to determine the working principles of this photocatalytic system. The correlation of these results allowed us to determine the pH-dependent rate-limiting steps in the photocatalytic system and give a rational explanation for the observed optimal activity at pH 4.5, as well as to provide a timescale for the electron transfer (ET) reactions between the sacrificial electron donor, the dye and the catalyst. Experimental details are described in the ESI.†

## Results and discussion

**Fig. 2:**
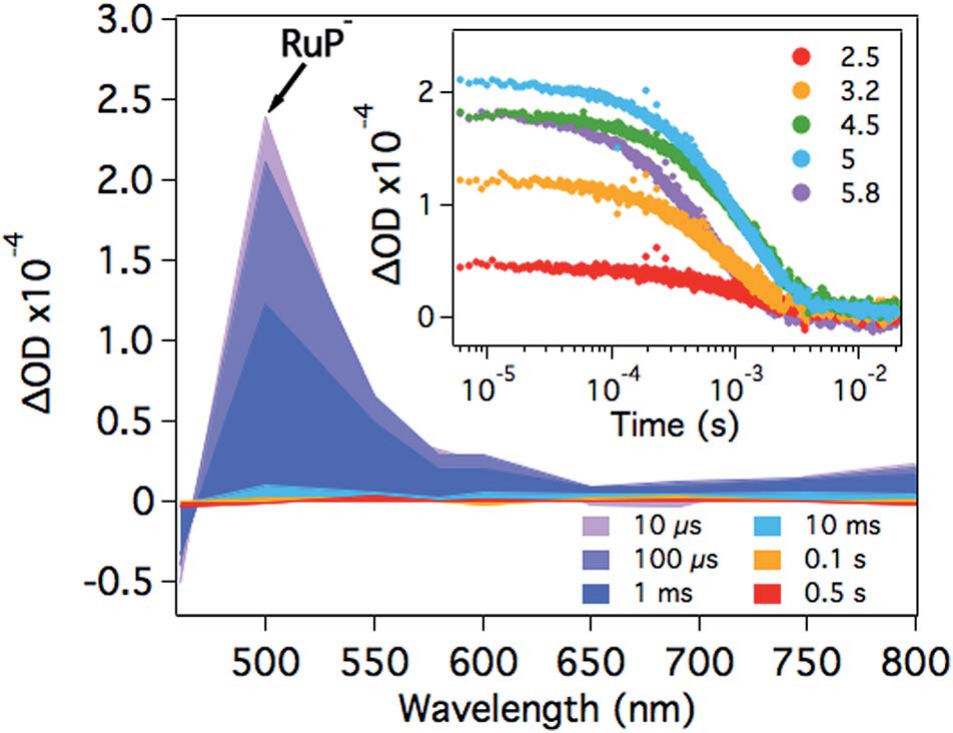
Transient absorption spectra of **RuP** (4 μM) in the presence of AA (0.1 M) at pH 4.5 as a function of time delay. The inset shows the corresponding kinetics probed at *λ* = 500 nm in the pH range between 2.5 and 5.8. The samples were excited at *λ* = 355 nm.

At pH 4.5, photoexcitation of **RuP** in the presence of AA leads to the efficient formation of **RuP^–^** within *t*_50%_ ∼ 250 ns through a reductive quenching mechanism, with a quantum yield estimated from transient emission studies of approximately 70%.^17^ The reduced photosensitiser **RuP^–^** shows a transient absorption peak at *λ* = 500 nm with a lifetime (*t*_50%_, calculations detailed in Fig. S1†) of 500–700 μs (Fig. 2).^35^ The yield of **RuP^–^** produced at different pH values can be determined from the initial amplitude (at ∼10 μs) of this **RuP^–^** transient absorption signal at *λ* = 500 nm. It is apparent (Fig. 2, inset) that this assay of the yield of **RuP^–^** increases with increasing pH, reaching a maximum at pH = 5. This behaviour can be explained by the different reactivity of two protonation states of ascorbic acid present in the pH range studied herein. At low pH, ascorbic acid exists primarily in its undissociated form H_2_A (p*K*_a_ = 4.17), whereas the monoprotic ascorbate anion (HA^–^) predominates at higher pH values (p*K*_a_ = 11.57). The ascorbate anion is a stronger reducing agent than its protonated form, and thus the reductive quenching of the excited dye, RuP*, is favoured at pH >4, where HA^–^ is the dominating species.^36–38^

**Fig. 3:**
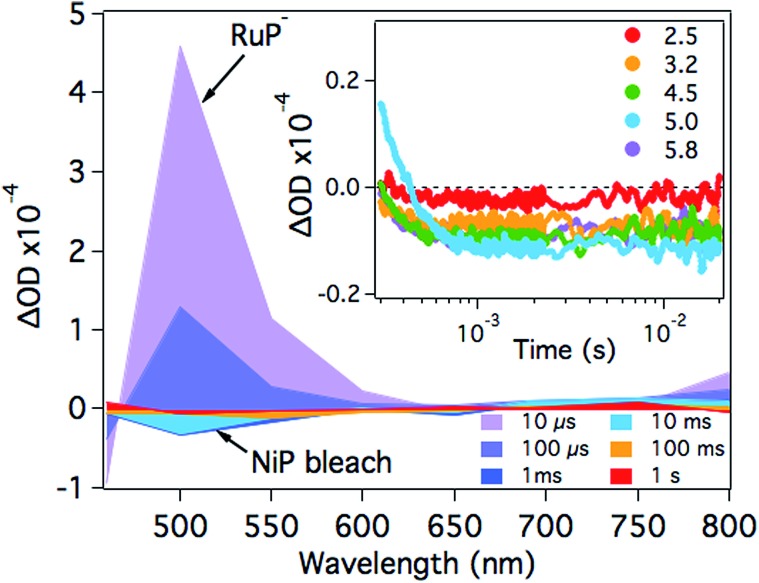
Transient absorption spectra of a **RuP** (4 μM) and **NiP** (8 μM) mixture in the presence of AA (0.1 M) at pH 4.5 as a function of time delay. The inset shows the time profile of the negative signal monitored at *λ* = 500 nm, assigned to the loss of ground state absorption of **NiP** in the pH range studied. The samples were excited at *λ* = 355 nm.

After the formation of **RuP^–^**, electrons should be transferred from the reduced dye to the catalyst. In the presence of **NiP**, the positive transient absorption signal corresponding to **RuP^–^** absorption at *λ* = 500 nm is rapidly quenched (within 50–100 μs on the range of pH values studied herein), leading to the appearance of a negative signal at longer timescales (500 μs to 1 s; Fig. 3 and S2†). This negative signal is assigned to electron transfer from **RuP^–^** to **NiP**, resulting in bleaching of ground state absorption of **NiP**.^17^ This bleach is not observed in the absence of either **RuP** or **NiP** (see for example Fig. 2 and S3†), suggesting that it is due to intermolecular electron transfer (ET) between **RuP^–^** and **NiP** (rather than the direct photoexcitation of **NiP**). The fast electron transfer kinetics between **RuP^–^** and **NiP** at all studied pH values suggests that this process is not limiting the catalytic activity of **NiP** (Fig. S2†). However, the long-lived transient absorption bleach signal corresponding to reduced **NiP** indicates that the subsequent protonation step is more likely to be the rate limiting reaction. We can also estimate the yield of **NiP** reduced by **RuP^–^** from the amplitude of the bleach (Fig. 3 inset). Thus, a greater negative signal indicates the reduction of more **NiP** due to ET from **RuP^–^**. It is apparent that the yield of reduced **NiP** increases as the pH is increased, reaching a maximum at pH = 5.

**Fig. 4:**
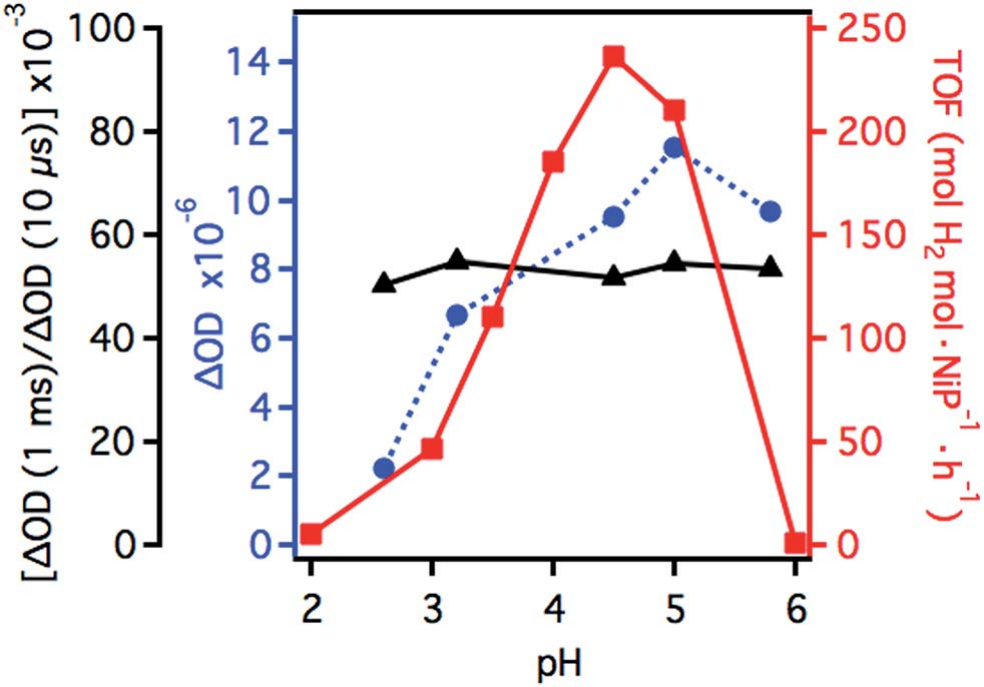
TOF_NiP_(H_2_) of a homogeneous AA (0.1 M) aqueous solution at different pH values, containing **RuP** (0.3 μmol, 133 μM) and **NiP** (0.1 μmol, 44 μM; red squares). Transient absorption signal amplitudes of the **NiP** bleach at 1 ms (absolute values, blue circles) and transient absorption amplitude ratios of **NiP** at 1 ms and **RuP^–^** at 10 μs (black triangles).

Fig. 4 compares the pH dependence of the 500 nm transient absorption bleach signal assigned to the yield of reduced **NiP** (blue circles) and the TOF_NiP_(H_2_) per catalyst molecule of the system (red squares) determined from bulk photocatalysis experiments reported previously (see ESI†).^17^ Also shown in Fig. 4 is the ratio of reduced **NiP** per **RuP^–^** (black triangles, calculations detailed in the ESI†). It is apparent that whilst both the TOF and yield of reduced **NiP** are strongly pH dependent, the ratio of reduced **NiP**/**RuP^–^** is independent of pH. Thus, our results suggest that the yield of reduction of **NiP** by **RuP^–^** is pH independent. In contrast, from pH 2 to 4.5, both the **NiP** reduction yield and the TOF_NiP_ increase. As the efficiency of electron transfer from **RuP^–^** to **NiP** is pH independent, the increase in the yield of reduced **NiP** with higher pH can be assigned directly to the increased efficiency of **RuP^–^** formation due to the pH dependence of the electron donating function of the ascorbic acid as discussed above. It is also striking from Fig. 4 that at pH >4.5, the TOF_NiP_ rapidly decreases despite the yield of reduced **NiP** remaining high. Such a sharp maximum in the pH dependence of TOF_catalyst_ has also been observed in many other photocatalytic systems.^14,15,17,25,39,40^

As the yield of reduced catalyst is approximately constant between pH 4.5 and 6, the drop on hydrogen generation towards neutral pH is strongly indicative of a decreasing activity in proton reduction catalysed by **NiP**. The exact catalytic mechanism for proton reduction using nickel bis(diphosphine) catalysts is still not fully elucidated, with little evidence of the catalytic intermediates in aqueous media.^41,42^ Although protonation of the reduced Ni species may in principle occur at the pendant amines of the ligand or directly at the Ni metal centre, DFT calculations support protonation of the amines.^41^ This agrees with the dependence of the electrocatalytic activity on acid concentration of bis(diphosphine) nickel electrocatalysts, which has been explained by the presence of pendant amines in the second coordination sphere. These amines with a relatively low p*K*_a_ have been suggested to act as proton relays between the solvent and the metal centre.^13,18,19,43^ Although these studies were mainly performed in pure organic solvents or aqueous-organic solvent mixtures in the presence of strong acids, the electrocatalytic proton reduction activity of **NiP** was observed to increase towards more acidic pH.^17^ In this article, we detail the dependence of the catalytic activity of **NiP** on pH in pure water.

**Fig. 5:**
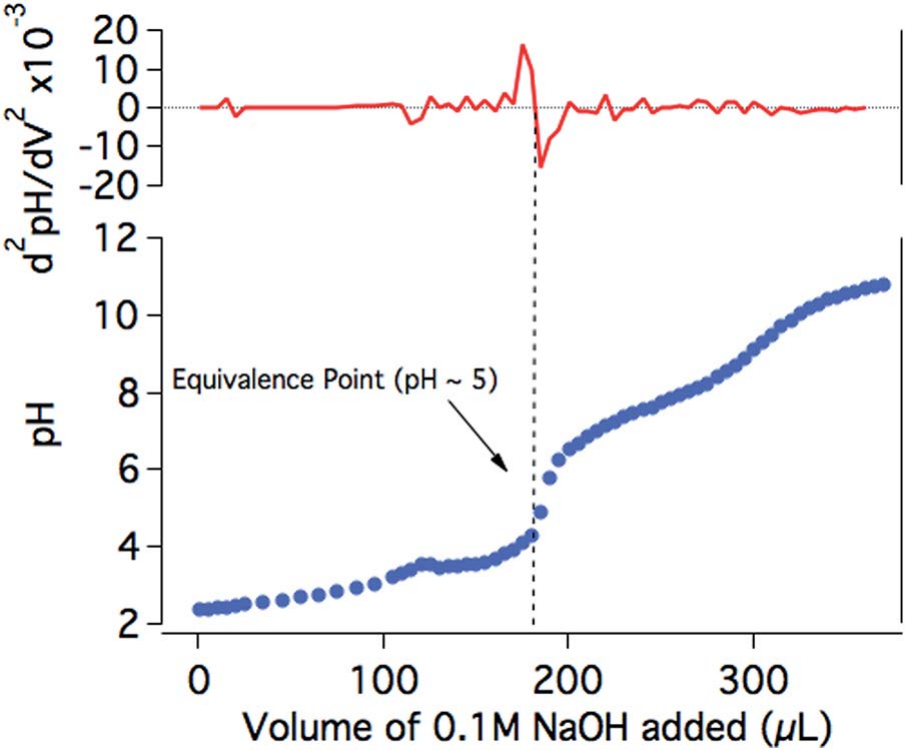
Titration of **NiP** (0.57 mM) in KCl (0.1 M) with NaOH (0.1 M; blue trace) and the second derivative of the pH with respect to the added volume (red trace). **NiP** is less soluble below pH 3 and dissolves completely upon the addition of approximately 120 μL of NaOH.

In order to further investigate the drop in the H_2_ production yield of the photocatalytic system towards neutral pH, the protonation state of **NiP** at different pH values was studied. The titration of **NiP** with NaOH (0.1 M) shows two equivalence points, at pH ∼5 and pH ∼9 (Fig. 5). In agreement with previous reports, these processes are assigned to the deprotonation of the pendant amines and the second deprotonation of the phosphonic acid groups, respectively.^20,44^ The assignment of the deprotonation of the amines is further confirmed by the presence of only one equivalence point at pH ∼5 for the titration of an analogous bis(diphosphine) nickel complex where the phopsphonic acid substituents are protected with ethyl ester groups (**NiP^Et^**) (Fig. S4†). A p*K*_a_ ∼3 is calculated from the Henderson–Hasselbach equation for the pendant amines in the ligand with an equivalence point at pH ∼5 (see ESI† for details), meaning that at pH >5, the amines are largely deprotonated. Since these amines are considered to play an important role as proton relays between the solvent and the nickel metal centre,^18,19^ it is likely that, at less acidic media, the catalytic efficiency is limited by a poor degree of protonation of the pendant amines of the catalyst, which inhibits the ability of **NiP** to reduce protons to H_2_. It is worth noting that the photosensitiser employed in our studies contains phosphonic acid substituents. This dye was chosen for consistency and to allow for direct comparison with our previous studies.^17^ The p*K*_a_ values of **RuP** have been reported to be ∼1 and ∼12, suggesting that the buffer capacity of **RuP** within the pH range employed in this study is limited.^45,46^

Our results match well with the strong pH-dependencies reported with other proton reduction photocatalytic systems that employ either AA, triethanolamine (TEOA) or ethylenediaminetetraacetic acid (EDTA) as sacrificial electron donors.^14,15^ In acidic media, the sacrificial electron donor molecules become protonated, resulting in a poor electron-donating ability due to the anodic shift of the reduction potential.^4,17^ Hence our studies show that the optimum pH of active homogeneous proton reduction systems is a compromise between electron donating ability of the sacrificial agent and the optimum working environment for the catalyst.

## Conclusions

In summary, we have used transient absorption spectroscopy, combined with titration studies, electrochemistry and bulk photocatalytic experiments, to study the pH-dependence of the electron transfer reactions of a ruthenium-based photosensitiser and a nickel bis(diphosphine) catalyst for the production of H_2_ under visible light irradiation. Our results suggest that the yield and kinetics of the electron transfer from the sensitiser to the catalyst are independent of the pH. However, at pH <4.5, the catalysis is limited by the number of **RuP^–^** molecules available to reduce the catalyst due to the poor reducing character of undissociated AA. In contrast, at less acidic pH, low TOF_NiP_(H_2_) are observed despite the large concentration of **RuP^–^** molecules available to reduce **NiP**. Titration studies of **NiP** with NaOH show that at pH >5, the amines are largely deprotonated and electrochemical studies confirm the lower activity at such pH values.^17^ Since these amines have been reported to play an important role as proton relays between the solvent and the nickel metal centre, it is likely that the catalytic efficiency is limited by the lack of protonated amines in the nickel catalyst. In the wider context, our studies suggest that the pH of photocatalytic systems using a sacrificial agent has to be adjusted to match the pH at which the dye is effectively reduced by the sacrificial electron donor and the pH at which the catalyst can be efficiently protonated. We have also demonstrated how transient absorption spectroscopy, bulk photocatalytic and titration studies and electrochemical experiments can be combined for a rational analysis of limiting factors in a homogeneous photocatalytic system.

## Author contributions

E.P., A.R. and S.S. conducted the spectroscopic experiments. J.R.D. and A.R. designed the experiments. M.A.G. synthesised the compounds and carried out the titration experiments. M.A.G. and E.R. developed the photocatalytic system. E.P., A.R., M.A.G., E.R. and J.R.D. wrote the paper.
