# Supplementary material for: Unravelling the pH-dependence of a molecular photocatalytic system for hydrogen production
Source: Chem Sci. 2015 May 28;6(8):4855–9. doi: 10.1039/c5sc01349f (PMC5502398; doi:10.1039/c5sc01349f)
Supplement: Supplementary file 1 [file SC-006-C5SC01349F-s001.pdf]

Electronic Supplementary Material (ESI) for Chemical Science.
This journal is © The Royal Society of Chemistry 2015

Supporting Information

Unravelling the pH-dependence of a molecular photocatalytic system for hydrogen production

Anna Reynal,[a, b] Ernest Pastor,[a] Manuela A. Gross,[c] Shababa Selim,[a] Erwin Reisner,[c] and James. R. Durrant[a]

[a] Department of Chemistry, Imperial College London, Exhibition Road, London SW7 2AZ, UK.

[b] School of Chemistry, Newcastle University, Edward's Walk, Newcastle Upon Tyne, NE1 7RU, UK.

[c] Christian Doppler Laboratory for Sustainable SynGas Chemistry, Department of Chemistry, University of Cambridge, Cambridge, CB2 1EW, UK.

Contents **Page**

Experimental details S2

Figures S1 – S4 S3

Additional references S4

EXPERIMENTAL DETAILS

Synthesis

Chemicals were purchased from commercial suppliers with the highest purity available and used without further purification. **RuP**, **NiP** and **NiP^{Et}** were synthesised and fully characterised as reported elsewhere.[1]

Transient absorption spectroscopy measurements

The microsecond-second transient absorption decays were measured using a Nd:YAG laser (Big Sky Laser Technologies Ultra CFR Nd:YAG laser system, 6 ns pulse width). The third harmonic of the laser, corresponding to 355 nm, at a frequency of 0.8 Hz, was used as excitation pulse. The excitation density was typically adjusted to 350 μJ cm^{-2}, unless otherwise stated. A liquid light guide with a diameter of 0.5 cm was used to transmit the laser pulse to the sample. The probe light source was a tungsten lamp (Bentham IL1 tungsten lamp), and the probing wavelength was selected by using a monochromator (OBB-2001 dual grating, Photon Technology International) placed prior to the sample. Transient absorption data was collected with a Si photodiode (Hamamatsu S3071). The information was passed through an amplifier box (Costronics) and recorded using a Tektronics TDS 2012c oscilloscope (microsecond to millisecond timescale) and a National Instruments (NI USB-6211) DAQ card (millisecond to second timescale). The decays observed are the average between 500 and 1000 averages laser pulses. The data was processed using home-built software based on Labview.

Transient absorption experiments were carried out in freshly prepared AA (0.1 M) aqueous solutions, with the pH carefully adjusted by the addition of NaOH (0.1 M) or HCl (0.1 M). The solutions were purged with N_2 for 15 min and kept in the dark to avoid degradation prior to the measurements.

In Figure 4 of the main text, the **NiP/RuP$^{–}$** ratio refers to the number of **NiP** molecules reduced per **RuP** molecule at different pH values. This ratio was calculated from the transient absorption decay of a 4 μM **RuP** and 8 μM **NiP** solution in 0.1M AA, by dividing the change in optical density (ΔOD) at λ = 500nm at 1 ms (assigned to the loss of ground state absorption of **NiP** after electron transfer) by the ΔOD at 10 μs (transient absorbance of **RuP$^{–}$** before electron transfer). Both signals were measured by exciting the samples at λ_{ex} = 355 nm (E_{pulse} = 350 μJ·cm^2) and probing at λ_{ex} = 500 nm.

Hydrogen evolution photocatalytic measurements

Samples for photocatalytic measurements were prepared as follows. A freshly prepared aqueous solution of AA was titrated to the desired pH by adding 0.1 M NaOH or 0.1 M HBF_4, and then diluted to a final concentration of AA of 0.1 M. **RuP** (0.3 μmol in water) and **NiP** (0.1 μmol in methanol) were added to 2.25 mL of AA, leaving a headspace volume of 5.59 mL in the photoreactor. The samples were prepared in air protected from light by an Al foil. The reaction vessel was sealed with a rubber septum and purged with N_2 containing 2% CH_4 (internal standard for quantification of H_2 in the headspace by gas chromatography). The irradiated cross-section of the solution in the vials used was approximately 3.33 cm^2. All samples were irradiated with a Solar Light Simulator (Newport Oriel, 100 mW cm^{-2}) equipped with an AM 1.5G filter, a 420 nm long pass UV filter and a water filter to remove IR irradiation. H_2 evolution was monitored using an Agilent 7890A Series gas chromatograph equipped with a 5 Å molecular sieve column and a thermal conductivity detector. The oven temperature was kept at 45 °C and N_2 was used as a carrier gas at an approximate flow of 3 mL min^{-1}.

Determination of the pK_a of NiP and NiP^{Et}

NiP (4.5 mg) was suspended in KCl (0.1 M, 5 mL) at pH 2.5, and sonicated for 5 min, after which the compound partially dissolved. The solution was titrated at 22 °C with aliquots of NaOH (0.1 M) while stirring. After each addition of base, the pH was read after 2-3 min waiting time to allow for equilibration. **NiP** was not completely soluble at pH 2.5, but dissolved entirely with increasing amounts of base at pH 3 – 3.5. **NiP^{Et}** (0.47 mM) in 5 mL KCl (0.1 M, 20% acetonitrile) was titrated with NaOH (0.1 M) in the same way as **NiP**. The pH in the mixed solvent was recorded against an aqueous reference electrode, and is reported without correction.[2]

Phosphonic acids typically have very low pK_a values for the first deprotonation, ranging from 1-3.[3-6] We therefore assume that the first deprotonation of all 4 phosphonic acid moieties present in **NiP** takes place before the deprotonation of the pendent amines, which are weaker acids. Due to the poorer solubility of **NiP** at low pH, the first deprotonation of the phosphonic acids could not be observed. We suspect that deprotonation of the phosphonic acids and hence ionisation of the complex increases the solubility of the catalyst significantly. Therefore, we assign the first equivalence point (pH ~ 5) observed in the titration of **NiP** with NaOH to the deprotonation of the pendent amine moieties. This assignment is in agreement with data reported previously[7] and supported by the titration curve obtained for the diethyl phosphonate ester analogue of **NiP** (**NiP^{Et}**) with NaOH (0.1 M) (Figure S4). For **NiP^{Et}**, a strong equivalence point at pH 5.7 was observed, supporting the assignment of deprotonation of the pendent amines at this pH, as the phosphonate esters are unaffected by pH changes. We have assigned the equivalence point observed in Figure 4 at pH ~9 to the second deprotonation of the phosphonic acids.[4]

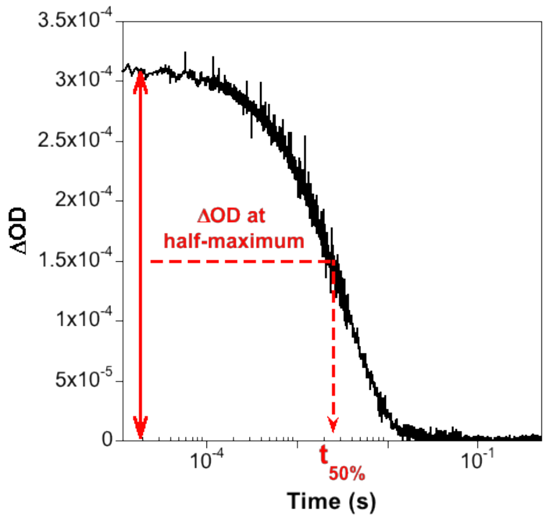

Figure S1. Transient absorption decay exemplifying the calculation of $t_{50\%}$ as the time required for the signal amplitude (ΔOD) to decrease half of its initial. $t_{50\%}$ is defined as the time required for the signal amplitude (ΔOD) to decrease half of its initial value (full width half maximum).

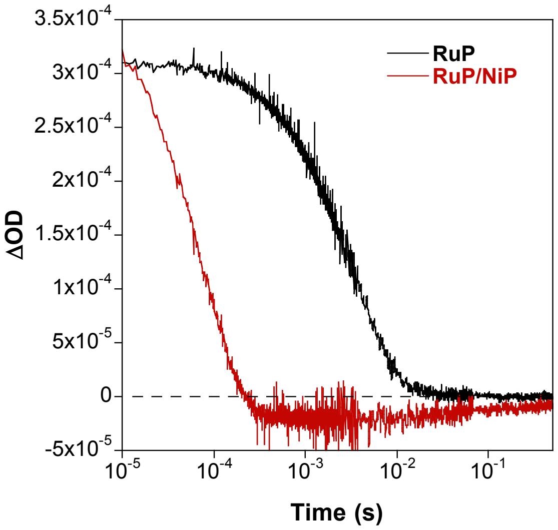

Figure S2. Transient decay of a solution of 4 µM **RuP** in 0.1M AA (black trace) and a solution of 4 µM **RuP** and 8 µM **NiP** in 0.1 M AA (red trace) at pH 4.5 after excitation with 355 nm light (350 µJ/cm^2) showing the fast ($t_{50\%}$ ~ 100 µs) electron transfer from **RuP**$^-$ to **NiP.**

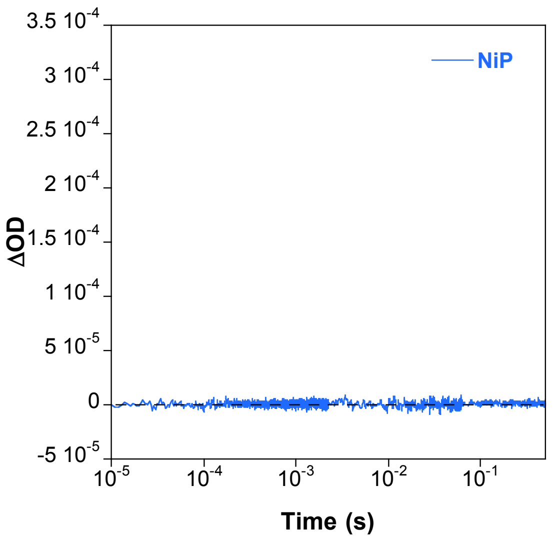

Figure S3. Transient absorption of a solution of 0.1 mM **NiP** in MeOH after excitation with 355 nm light (350 µJ/cm^2) showing no bleaching of the ground state of **NiP** in the absence of photosensitiser (**RuP)**.

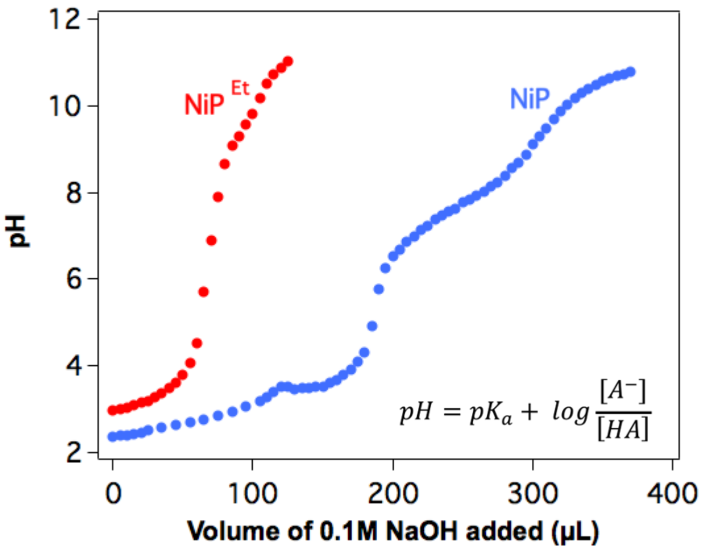

Figure S4. Titration of **NiP^{Et}** (0.47 mM) in KCl (0.1 M, 20% Acetonitrile) with NaOH (0.1 M; red trace) and **NiP** (0.57 mM) in KCl (0.1 M) with NaOH (0.1 M; blue trace). The pK$_a$ of the pendant amines calculated from the Henderson-Hasselbalch equation (inserted in graph) is pK$_a$ ~ 3.[8]

ADDITIONAL REFERENCES

1. M. A. Gross, A. Reynal, J. R. Durrant and E. Reisner, *J.Am.Chem.Soc.*, 2014, 136, 356-366.
2. S. Espinosa, E. Bosch and M. Rosés, *Anal. Chem.*, 2000, 72, 5193-5200.
3. K. Nagarajan, K. P. Shelly, R. R. Perkins and R. Stewart, *Can. J. Chem.*, 1987, 65, 1729-1733.
4. K. Swierczek, A. S. Pandey, J. W. Peters and A. C. Hengge, *J. Med. Chem.*, 2003, 46, 3703-3708.
5. M. S. Smyth, H. Ford Jr and T. R. Burke Jr, *Tetrahedron Lett.*, 1992, 33, 4137-4140.
6. N. Aliouane, S. Chafaa, T. Douadi, J.-J. Hélesbeux, M. A. Khan, O. Duval and G. Bouet, *Heteroat. Chem.*, 2010, 21, 51-62.
7. A. Dutta, S. Lense, J. Hou, M. H. Engelhard, J. A. S. Roberts and W. J. Shaw, *J.Am.Chem.Soc.*, 2013, 135, 18490-18496.
8. P. Atkins, J. De Paula, Elements of Physical Chemistry, 2013, 6th Ed, Oxford University Press.
